# Fbw7 regulates apoptosis in activated B-cell like diffuse large B-cell lymphoma by targeting Stat3 for ubiquitylation and degradation

**DOI:** 10.1186/s13046-016-0476-y

**Published:** 2017-01-10

**Authors:** Su Yao, Fangping Xu, Yu Chen, Yan Ge, Fen Zhang, Huijie Huang, Li Li, Danyi Lin, Xinlan Luo, Jie Xu, Donglan Luo, Xiaolan Zhu, Yanhui Liu

**Affiliations:** 1 Department of Pathology, Guangdong General Hospital & Guangdong Academy of Medical Sciences, Guangzhou, Guangdong 510080 People’s Republic of China; 2 Department of Pathology, Dongguan People’s Hospital, Dongguan, Guangdong 523059 People’s Republic of China

**Keywords:** Fbw7, Stat3, DLBCL, Activated B-cell, Apoptosis, Ubiquitylation

## Abstract

**Background:**

The ubiquitin-ligase Fbw7 acts as a tumor suppressor, targeting lots of proto-oncogenes for proteolysis. However, the exact role of Fbw7 in diffuse large B-cell lymphoma (DLBCL) development remains unclear.

**Methods:**

We evaluated Fbw7 expression in patient samples of DLBCL using immunohistochemical staining. The effect of Fbw7 overexpression on cell viability and apoptosis was investigated using activated B-cell (ABC) like DLBCL cell lines. The mechanism of Fbw7 activity in DLBCL was investigated using immunoprecipitation, ubiquitination, western blot and qualitative analyses.

**Results:**

The non-germinal center B-cell-like subtype of DLBCL showed reduced Fbw7 expression compared with the germinal center B-cell (GBC) subtype, and low Fbw7 expression was associated with a worse prognosis. Fbw7 overexpression caused decreased cell viability and increased apoptosis rates in the ABC-DLBCL cell lines SU-DHL-2 and OCI-LY-3. Importantly, Stat3 and phospho-Stat3^Tyr705^ stability were reduced following Fbw7 overexpression in ABC-DLBCL cell lines. In HEK293T and SU-DHL-2 cells, we demonstrated that Fbw7 interacts with Stat3 and pStat3^Tyr705^ to regulate their ubiquitylation and degradation. Downstream anti-apoptotic target genes of activated Stat3, including Myc, Survivin, Mcl-1, Pim-1, Bcl-2 and Bcl-xl showed decreased mRNA expression following exogenous Fbw7 overexpression. The negative relationship between Fbw7 and pStat3^Tyr705^ levels was also confirmed in DLBCL patient samples.

**Conclusion:**

The ubiquitin-ligase Fbw7 mediates apoptosis through targeting Stat3 for ubiquitylation and degradation in ABC-DLBCL. Thus, our study may offer a promising approach for ABC-DLBCL therapy through Stat3 inhibition.

**Electronic supplementary material:**

The online version of this article (doi:10.1186/s13046-016-0476-y) contains supplementary material, which is available to authorized users.

## Background

Diffuse large B-cell lymphoma (DLBCL) is the most common type of non-Hodgkin lymphoma in adults, contributing to nearly 40% of new diagnoses [1]. However, DLBCL is involuted both in clinical presentation and morphology. To resolve this problem, DLBCL is now classified as GCB-DLBCL and ABC-DLBCL by genetic profiling [2, 3], and patients with the ABC subtype show a significantly poorer outcome compared with the GCB subtype [4]. Immunohistochemistry (IHC) for CD10, Bcl-6 and MUM1 can also differentiate between the GCB and non-GCB subtypes of DLBCL and predicts similar outcomes to genetic profiling [5]. Improved therapies are required for all patients with DLBCL but most urgently for those with the ABC subtype, which is the most chemoresistant and has a worse prognosis [6].

ABC-DLBCL is associated with many different oncogenic events, but all ultimately act on nuclear factor-κB (NF-κB) to promote lymphomagenesis [7]. Therefore, signal transducer and activator of transcription 3 (Stat3), which cooperates with NF-κB signaling, is a promising candidate for ABC-DLBCL targeted therapy [8, 9]. Stat3 is constitutively activated in various tumor types, contributing to enhanced proliferation, survival, angiogenesis and immune evasion via several mechanisms [10–14]. Constitutively activated Stat3 in ABC-DLBCL is associated with poor survival [15]; moreover, Stat3 activation is a biomarker for poor survival in DLBCL after rituximab plus cyclophosphamide, doxorubicin, vincristine, and prednisone (R-CHOP) treatment [16].

Fbw7, also known as CDC4, is a substrate recognition element of the evolutionarily conserved SCF-type ubiquitin ligase complex. Acting as a tumor suppressor in human cancer, Fbw7 substrates include several proto-oncogenes, which are ubiquitylated and tagged for proteasomal degradation [17–26]. Fbw7 genetic deletion results in developmental defects, embryonic lethality and genetic instability, and Fbw7 inactivation by loss of expression or mutation is associated with tumor development [27]. In the hematopoietic system, Fbw7 inactivation leads to hematopoietic stem cell (HSC) depletion by active cell cycling, which can initiate leukemia [28, 29]. Another study showed that HSC differentiation was mediated by Fbw7 regulating Myc stability [30]. Although Fbw7 targets various substrates for degradation that have crucial roles in cell cycle, apoptosis and differentiation, the role of Fbw7-mediated degradation of such targets remains unclear.

Here, we investigate Fbw7 expression in DLBCL. Interestingly, Fbw7 showed lower expression in the non-GCB-DLBCL subtype compared with GCB-DLBCL. The major phenotype associated with Fbw7 overexpression in ABC-DLBCL cell lines was the regulation of cell apoptosis. Furthermore, we confirmed that Fbw7 targets Stat3 and pStat3^Tyr705^ for ubiquitin-dependent degradation., and the downstream anti-apoptosis target genes of activated Stat3, including Pim1, Survivin, Mcl-1, Myc, Bcl2 and Bcl-xl showed reduced mRNA expression following exogenous Fbw7 overexpression, in a cell-dependent manner. Together, these results reveal an Fbw7-dependent mechanism that regulates Stat3 ubiquitylation and degradation, providing new insight into the tumor suppressor role of Fbw7, and suggesting that Fbw7 may offer a promising new approach to ABC-DLBCL therapy.

## Methods

### Reagents

The following antibodies were used in the study: anti-Fbw7 (for IHC) (H00055294-M02, Abnova, Taipei City, Taiwan), anti-Fbw7 (for western blots) (ab109617, Abcam, Cambridge, UK), anti-Stat3, 12640; anti-phospho-Stat3^Tyr705^, 4113; anti-Ubiquitin, 3936 (Cell Signaling Technology, Beverly, MA, USA), anti-Myc tag, 16286–1-AP; anti-Flag tag, 66008–2-Ig; and anti-β-actin, 60008–2-Ig; anti-Myc, 10828–1-AP; anti-Notch, 10062–2-AP; anti-Jun, 10024–2-AP; anti-DEK, 16448–1-AP; anti-Mcl1, 16225–1-AP(Proteintech, Rosemont, IL, USA). All other chemicals were purchased from Sigma-Aldrich (St. Louis, MO, USA) and Amresco (Dallas, TX, USA).

### Cell culture

The DLBCL cell lines SU-DHL-2 and OCI-LY-3 were cultured in RPMI-1640 supplemented with 10% fetal bovine serum (FBS; Gibco, Carlsbad, CA, USA), and HEK293T cells were cultured in DMEM supplemented with 10% FBS. Cells were maintained in a humidified chamber with 5% CO_2_ at 37 °C. The identity of cell lines was confirmed by short tandem repeats-polymerase chain reaction (STR-PCR) genotyping.

### Cell transfection

Vectors containing Fbw7, Stat3 and Ubiquitin were generated by cloning PCR amplified full-length human cDNAs into pcDNA3.1. And the human Stat3 siRNA target sequence was 5′-CACAT GCCAC TTTGG TGTTT CATAA-3′. Lipofectamine 3000 (Invitrogen, Carlsbad, CA, USA) was using to perform transfections according to the manufacturer’s instructions.

### Tissue samples and IHC

From 2005 to 2011, 165 human DLBCL samples were collected at the Guangdong General Hospital. The study was approved by Guangdong general hospital Biomedical Research Ethics Committee, and written informed consent was obtained from all the patients. Patients with DLBCL have been followed up for at least 5 years with a intervals of 1–3 months until July 2016. The clinicopathological characteristics of the DLBCL patients are showed in Additional files 1 and 2.

Samples were probed using the indicated antibodies. Paraffin-embedded samples were made into a tissue microarray. Staining was evaluated by two blinded individuals, and the scoring criteria was: 0 (no staining), 1 (weak staining), 2 (intermediate staining) and 3 (strong staining). Two pathologist gave a score and the final score was the average.

### Apoptosis analysis

Cells were first transfected with an Fbw7 expression plasmid or vector control, and then Doxorubicin (MP Biomedicals, Illkirch-Graffenstaden France) was used to induce apoptosis. We used the Annexin V-FITC Apoptosis Detection Kit (KeyGEN BioTECH, Nanjing, China), according to the manufacturer’s instructions, and the percentage of apoptotic cells was detected by flow cytometry analysis.

### Cell viability assay

For cell viability assay, 5 × 10^4^ cells per well were plated in 96-well, and then incubated with the appropriate medium containing Doxorubicin for 24 h. Assays were performed using the Cell Titer-Glo Luminescent Cell Viability Assay kit (Promega, Madison, WI, USA), according to the manufacturer’s instructions.

### Western blotting

Cells were lysed in RIPA lysis buffer (0.1% SDS, 50 mM Tris containing 150 mM NaCl, 1% Triton X-100 and 1% sodium deoxycholate; pH 7.2) with cocktails inhibitor of protease and phosphatase (Merck, Kenilworth, NJ, USA) on ice for 30 min and centrifuged at 14 000 × *g* for 30 min. According to the protein concentration of BCA Assay (Pierce, Rockford, IL, USA), 40 μg of protein was loaded on 8% SDS-PAGE gels. And then protein was transferred to PVDF membranes (Millipore, Billerica, MA, USA). Following transfer, blots were blocked, incubated with primary and secondary antibodies and exposed to film using standard procedures.

### Immunoprecipitation and ubiquitination assay

Cells were lysed in RIPA lysis buffer, and the lysates were immunoprecipitated with the indicated antibodies on protein A/G beads (Millipore) overnight. The beads were then washed and boiled in SDS loading buffer. Immunoprecipitated protein complexes were assessed using Western blotting. To detect ubiquitination of Stat3 and pStat3^Tyr705^, 10 mM N-ethylmaleimide was added in the lysis buffer.

### RNA extraction and qPCR analysis

Total RNAs were purified using RNAiso Plus, and first-strand cDNA was generated with PrimeScript RT Master Mix (Takara, Shiga, Japan). qPCR was carried out using SYBR Premix Ex Taq (Takara) on an ABI 7500 PCR system (Applied Biosystems, Carlsbad, CA, USA). The PCR protocol was made up of 40 cycles of clocking at 95 °C for 5 s and 60 °C for 30 s. The data was represented relative to β-actin, calculated using the 2^−ΔΔCT^ method. The primers for PCR reactions are listed in Additional file 3.

### Statistical analysis

Statistical analyses were carried out using the SPSS 16.0 statistical software (SPSS Inc., Chicago, IL, USA). Data are shown as mean ± SD. The relationships between Fbw7 expression and other clinicopathological factors were determined using Pearson *χ* ^2^ test. Kaplan-Meier survival analysis was applied to illustrate the outcome relevance of Fbw7 in univariate analysis. Each assay was performed in three repeated experiments. The Student *t* test was used to compare two groups of independent samples. Correlations between Fbw7 and pStat3^Tyr705^ levels were confirmed using the Spearman rank correlation. Values of *P <* 0.05 were considered significant.

## Results

### Decreased Fbw7 expression in non-GCB DLBCL compared with GCB-DLBCL is significantly correlated with poor survival

**Fig. 1:**
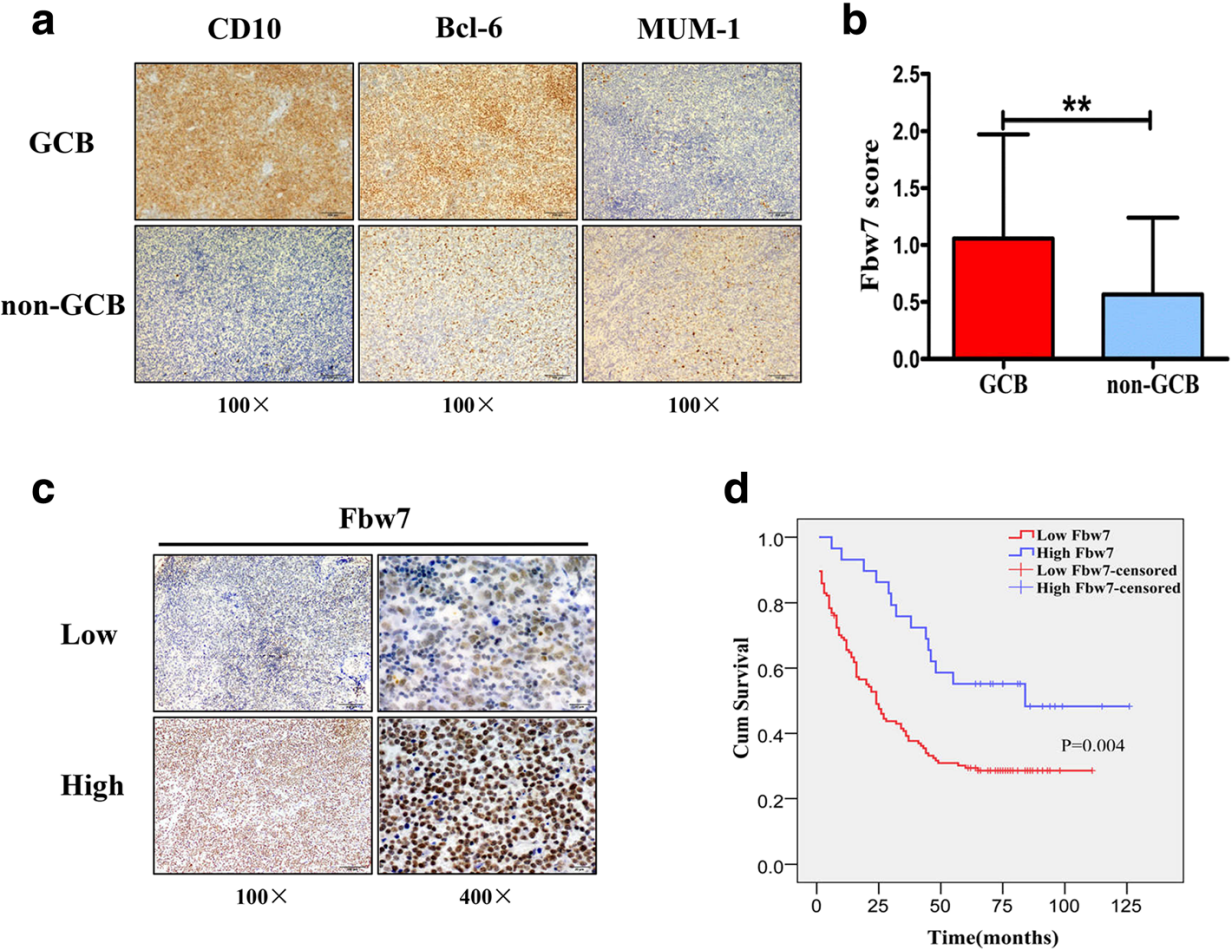
Fbw7 shows reduced expression in non-GCB DLBCL. **a** for 165 cases of DLBCLs, the classification of GCB and non-GCB groups was previously confirmed by clinical IHC of CD10, Bcl-6 and MUM1; representative IHC cases from GCB and non-GCB patients are shown. **b** bar chart of Fbw7 expression in the GCB (*n* = 43) and non-GCB (*n* = 122) groups. Statistical significance was determined by a two-tailed, Student *t* test. **, *P <* 0.01. **c** IHC staining of Fbw7 in DLBCL, “score ≤ 1” was defined as the low expression group and “score > 1” was defined as the high expression group. Low Fbw7 expression and high Fbw7 expression cases were shown. **d** Kaplan-Meier survival curve shows a significant association between low Fbw7 levels and poor survival in DLBCL patients (*P* = 0.004). Low Fbw7 (*n* = 30), high Fbw7 (*n* =134) and missing follow-up (*n* = 1)

To investigate the role of Fbw7 in DLBCL, Fbw7 expression was first analyzed in DLBCL through IHC. Using the Hans program [5], 165 patients were divided into two groups (non-GCB and GCB) based on CD10, Bcl-6 and MUM1 expression by IHC during the clinical diagnosis of pathology; representative IHC images of GCB and non-GCB are shown (Fig. 1a). IHC showed that Fbw7 expression was decreased in the non-GCB group compared with the GCB group (*P <* 0.01; Fig. 1b). Low Fbw7 expression and high expression cases were shown (Fig. 1c). The correlation between Fbw7 expression and other clinicopathological features, such as patient sex, age, EB virus status and tumor stage showed no significant differences (P > 0.05; Additional file 1). Kaplan-Meier analysis indicated that patients with low Fbw7 expression showed a significantly worse outcome compared with those with high Fbw7 expression (Fig. 1d). The median survival time of DLBCL patients with low Fbw7 expression was 44 months, which was significantly shorter than those with high Fbw7 expression (81 months). Together, these results clearly show that Fbw7 expression is decreased in non-GCB-DLBCL compared with GCB-DLBCL, and low Fbw7 expression is associated with a worse prognosis.

### Fbw7 regulates apoptosis in ABC-DLBCL

**Fig. 2:**
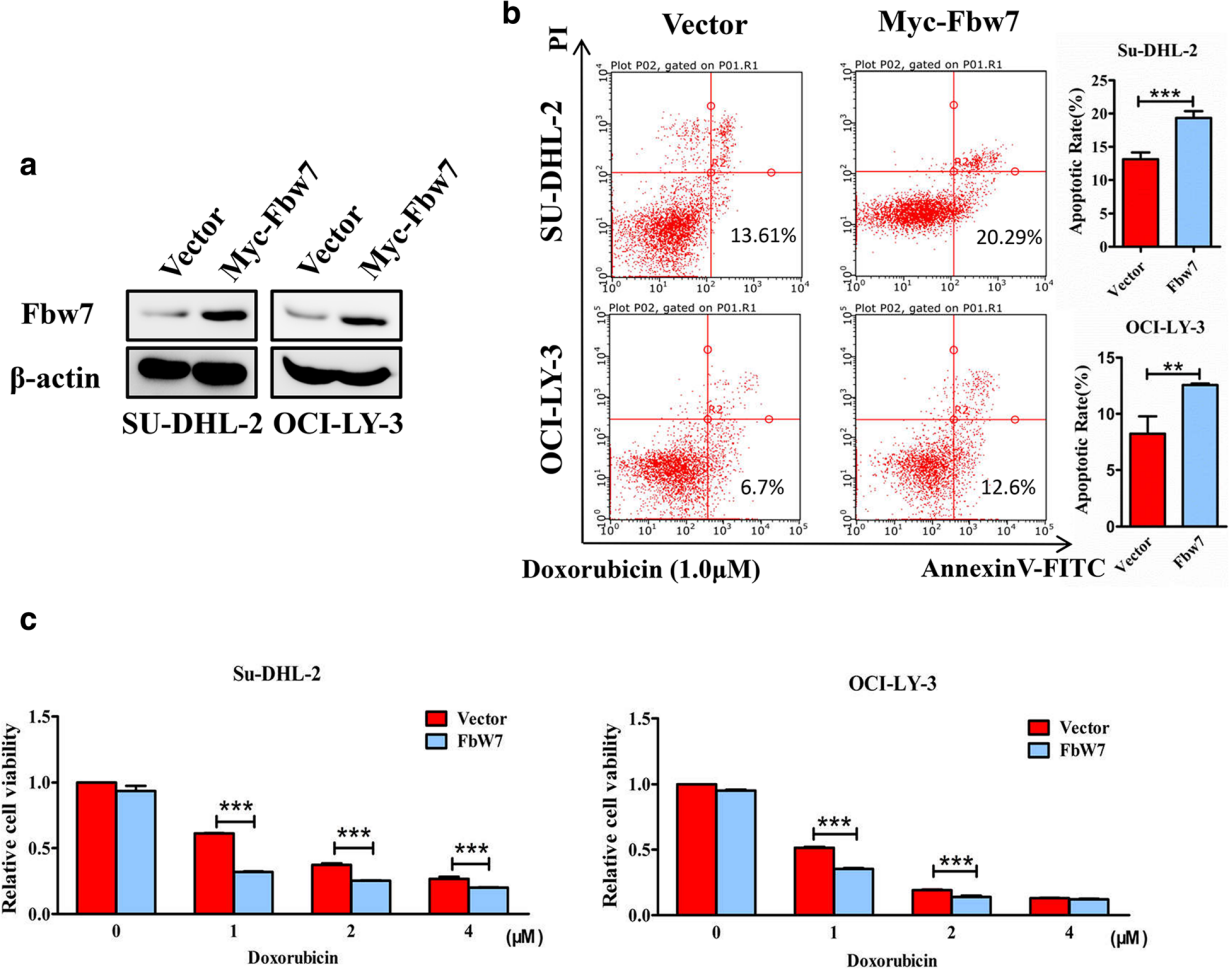
Fbw7 overexpression promotes apoptosis in ABC-DLBCL. **a** Western blot showing increased Fbw7 expression in the ABC-DLBCL cell lines SU-DHL-2 and OCI-LY-3 after transfection with Fbw7 expression plasmid for 48 h. **b** Annexin-V–PE staining, followed by flow cytometry analysis to detect the percentage of apoptotic cells. In the indicated ABC-DLBCL cell lines, Fbw7 was upregulated by transfection (pcDNA3.1 vector was used as a negative control) for 24 h. Cell lines were cultured with Doxorubicin (1.0 μM) for 24 h to induce apoptosis before flow cytometry. Statistical significance was determined by a two-tailed, paired Student *t* test. **, *P <* 0.01; ***, *P <* 0.001. **c** Cell viability assays show that ABC-DLBCL cell lines were more sensitive to Doxorubicin after transfection with Fbw7 plasmid. ABC-DLBCL cells with 24 h exogenous Fbw7 expression were cultured with the indicated concentrations of Doxorubicin for 24 h before cell viability assays were performed. Statistical significance was determined by a two-tailed Student *t* test. ***, *P <* 0.001. Data are presented as mean ± SD from three independent experiments

Based on the Hans algorithm, IHC can be used to differentiate the GCB and non-GCB subtypes of DLBCL, and this predicts survival similar to genetic profiling (GCB and ABC subtypes). The prevalence of Fbw7 downregulation in non-GCB subtype raises an intriguing possibility that Fbw7 overexpression may be a tumor-inhibiting event in ABC-DLBCL. To test this possibility, the ABC-DLBCL cell lines SU-DHL-2 and OCI-LY-3 were transfected with an Fbw7 expression plasmid, and western blotting confirmed that Fbw7 expression was greatly increased after transfection (Fig. 2a). Next, we investigated how Fbw7 affects the apoptotic response using Doxorubicin to induce apoptosis. To detect the percentage of apoptotic cells, we stained with Annexin-V-PE followed by flow cytometry analysis. As predicted, SU-DHL-2 and OCI-LY-3 cell lines were more sensitive to apoptotic stimuli after transfecting an Fbw7 plasmid compared with vector (Fig. 2b). To investigate the tumor inhibitory effect of Fbw7 in ABC-DLBCL, cells were transfected with the Fbw7 plasmid or vector and treated with Doxorubicin at different concentrations. As expected, cell viability decreased significantly in the Fbw7 overexpression group compared with the vector group (Fig. 2c). Although it’s reported that Fbw7 also regulates proliferation by suppressing tumorigenesis in many cancers, our results of CCK8 assays and cell cycle showed no significant difference after Fbw7 overexpression in ABC-DLBCL cell lines (Additional file 4).

### Fbw7 regulates the stability of Stat3

**Fig. 3:**
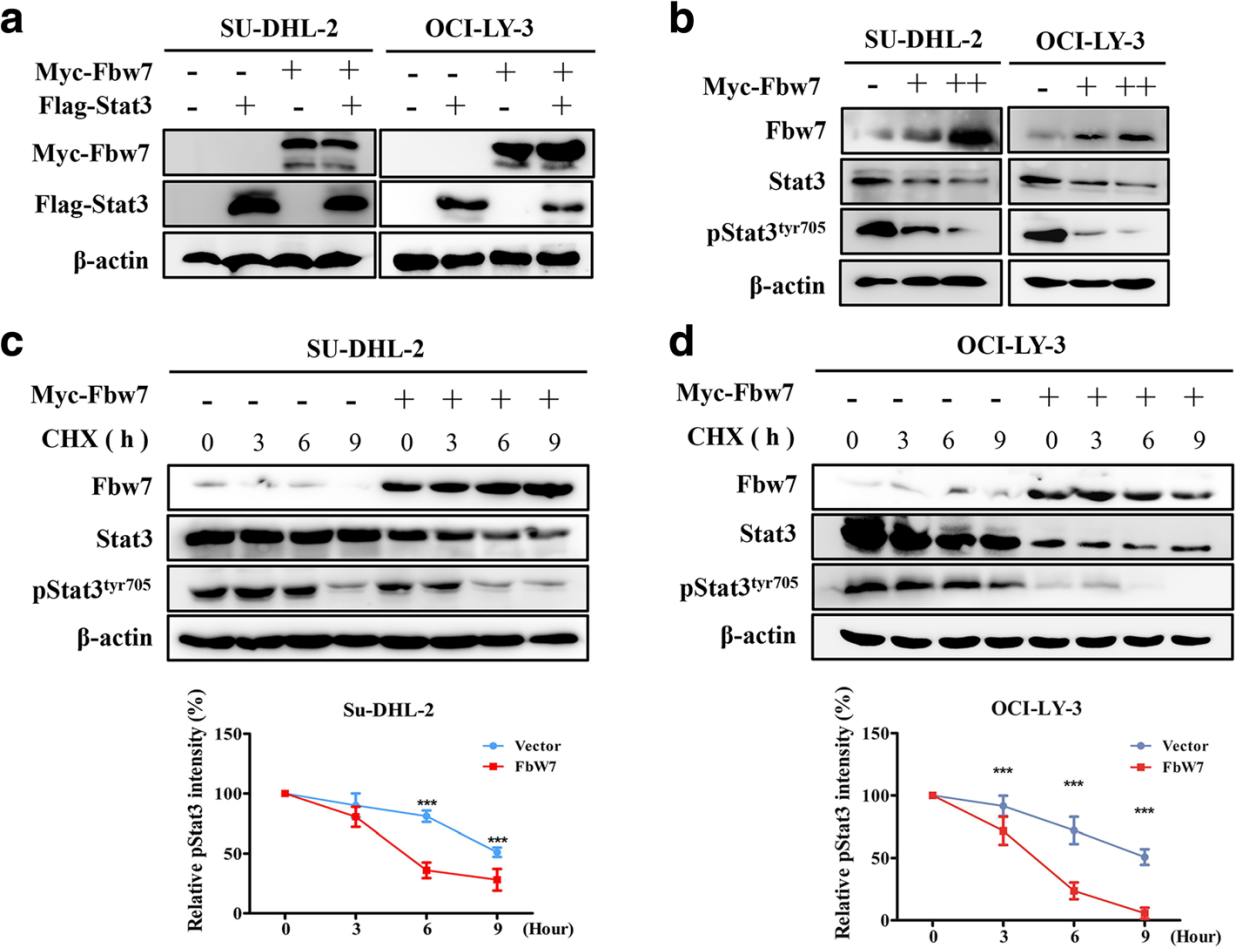
Fbw7 reduces the stability of Stat3. **a** western blotting of exogenous Stat3 in SUDHL-2 and OCI-LY-3 cells co-expressing Fbw7. **b** Fbw7 upregulation decreases endogenous Stat3 and especially pStat3^Tyr705^ expression. **c** and **d,** Fbw7 decreases the stability of Stat3 and pStat3^Tyr705^. Myc-Fbw7 was transfected into SUDHL-2 and OCI-LY-3 cells. After treating cells with cycloheximide (CHX; 10 mg/ml) for the indicated time intervals, Fbw7, Stat3 and pStat3^Tyr705^ levels were examined by immunoblotting (top). Western blots were quantified via densitometry, and the mean ratios of the indicated proteins from three independent experiments are shown at the bottom of the figure. Data are shown as mean ± SD in the line graph for three independent experiments. Statistical significance was determined by a two-tailed unpaired Student *t* test. ***, *P <* 0.001

Previous studies have shown that Stat3 is constitutive activated in ABC-DLBCL, causing us to evaluate whether Fbw7 regulates Stat3 expression or activity. SU-DHL-2 and OCI-LY-3 cells were transfected with Fbw7 and Stat3 plasmids, and western blotting indicated that Fbw7 decreased the exogenous Stat3 protein level (Fig. 3a). Similarly, Fbw7 overexpression markedly decreased endogenous Stat3 and pStat3^Tyr705^ levels in a dose-dependent manner (Fig. 3b). To investigate whether and how Fbw7 regulates Stat3 stability, SU-DHL-2 cells were treated with an inhibitor of protein synthesis cycloheximide (CHX), and the stability of endogenous Stat3 and pStat3^Tyr705^ was examined. The half-life of pStat3^Tyr705^ decreased from 9 h to approximately 5 h upon Fbw7 overexpression (Fig. 3c). Similarly, Fbw7 overexpression in OCI-LY-3 cells significantly reduced pStat3^Tyr705^ levels (Fig. 3d). These results reveal that Fbw7 reduced Stat3 signaling by promoting its degradation.

### Fbw7 targets Stat3 for ubiquitylation

**Fig. 4:**
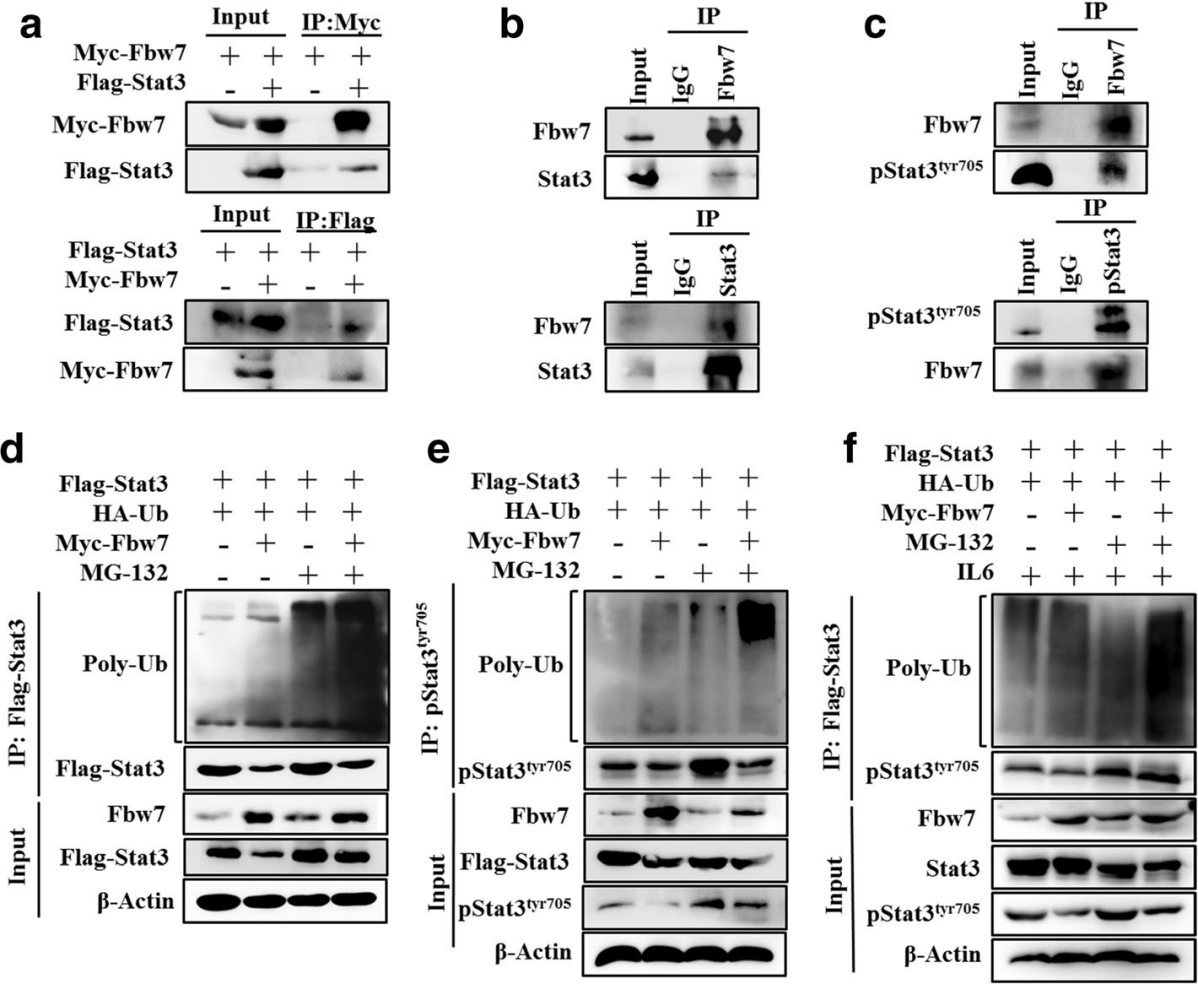
Fbw7 interacts with and ubiquitinates Stat3. **a** Fbw7 interacts with Stat3 at exogenous levels. Immunoblotting analysis of lysates after immunoprecipitation from HEK293T cells transfected with Myc-Fbw7 and Flag-Stat3. **b** Fbw7 interacts with Stat3 at endogenous levels. Cell lysates from SU-DHL-2 cells were immunoprecipitated with anti-Fbw7 or anti-Stat3 antibody, followed by immunoblotting with anti-Fbw7 or anti-Stat3 antibody, respectively. IgG was used as a control. **c** Fbw7 interacts with pStat3^Tyr705^ at endogenous levels. Cell lysates from SU-DHL-2 cells were immunoprecipitated with anti-Fbw7 or anti-pStat3^Tyr705^ antibody, followed by immunoblotting with anti-Fbw7 or anti- pStat3^Tyr705^ antibody, respectively. **d** and **e,** Flag-Stat3 and HA-ubiquitin were co-expressed with Myc-Fbw7 in HEK293T cells. After cells were treated with or without 10 mM MG132 for 6 h, Stat3 and pStat3^Tyr705^ were immunoprecipitated with anti-Flag or anti-pStat3^Tyr705^ antibody, and the polyubiquitination status of Stat3 and pStat3^Tyr705^ was detected by immunoblotting. **f** similar to E, cells were treated with 10 ng/L IL6 for 6 h to activate Stat3 signaling. The experiments were repeated three times, and representative images of blots are shown

Next, we investigated the ability of Fbw7 to interact with Stat3. Myc-Fbw7 and Flag-Stat3 were co-expressed in HEK293T cells. Co-immunoprecipitation analysis showed that Stat3 co-immunoprecipitated with Myc-Fbw7 by anti-Myc antibody (Fig. 4a, top). Similarly, immunoprecipitation of Flag-Stat3 by anti-Flag antibody led to co-immunoprecipitation of Myc-Fbw7 (Fig. 4a, bottom). Endogenous Fbw7 and Stat3 were immunoprecipitated from SU-DHL-2 cells and the presence of endogenous Stat3 and Fbw7 was detected, respectively (Fig. 4b). Similarly, endogenous Fbw7 and pStat3^Tyr705^ were immunoprecipitated from SU-DHL-2 cells, and the presence of endogenous pStat3^Tyr705^ and Fbw7 was detected, respectively (Fig. 4c). Similar results were obtained in HEK293T cells (Additional file 5). Together, these results show that Fbw7 can interact with Stat3 and pStat3^Tyr705^.

Fbw7 is a ubiquitin-ligase that targets several proteins for ubiquitination and degradation. Therefore, we then speculated that Fbw7 might directly regulate Stat3 stability through this activity. To assess this possibility, Flag-Stat3 and HA-ubiquitin were co-expressed with and without Myc-Fbw7 in HEK293T cells. Immunoblotting showed that ubiquitination of Stat-3, and especially pStat3^Tyr705^, strongly increased after Fbw7 expression in the presence or absence of MG132, an inhibitor of the 26S proteasome (Fig. 4d and e). Immunoblotting showed similar results for pStat3^Tyr705^ ubiquitination after cells were treated with IL-6 cytokines (Fig. 4e). These results conclusively indicate that Fbw7 regulates Stat3 protein levels through ubiquitination and proteasomal degradation.

### Fbw7 inhibits apoptosis regulators downstream of Stat3

**Fig. 5:**
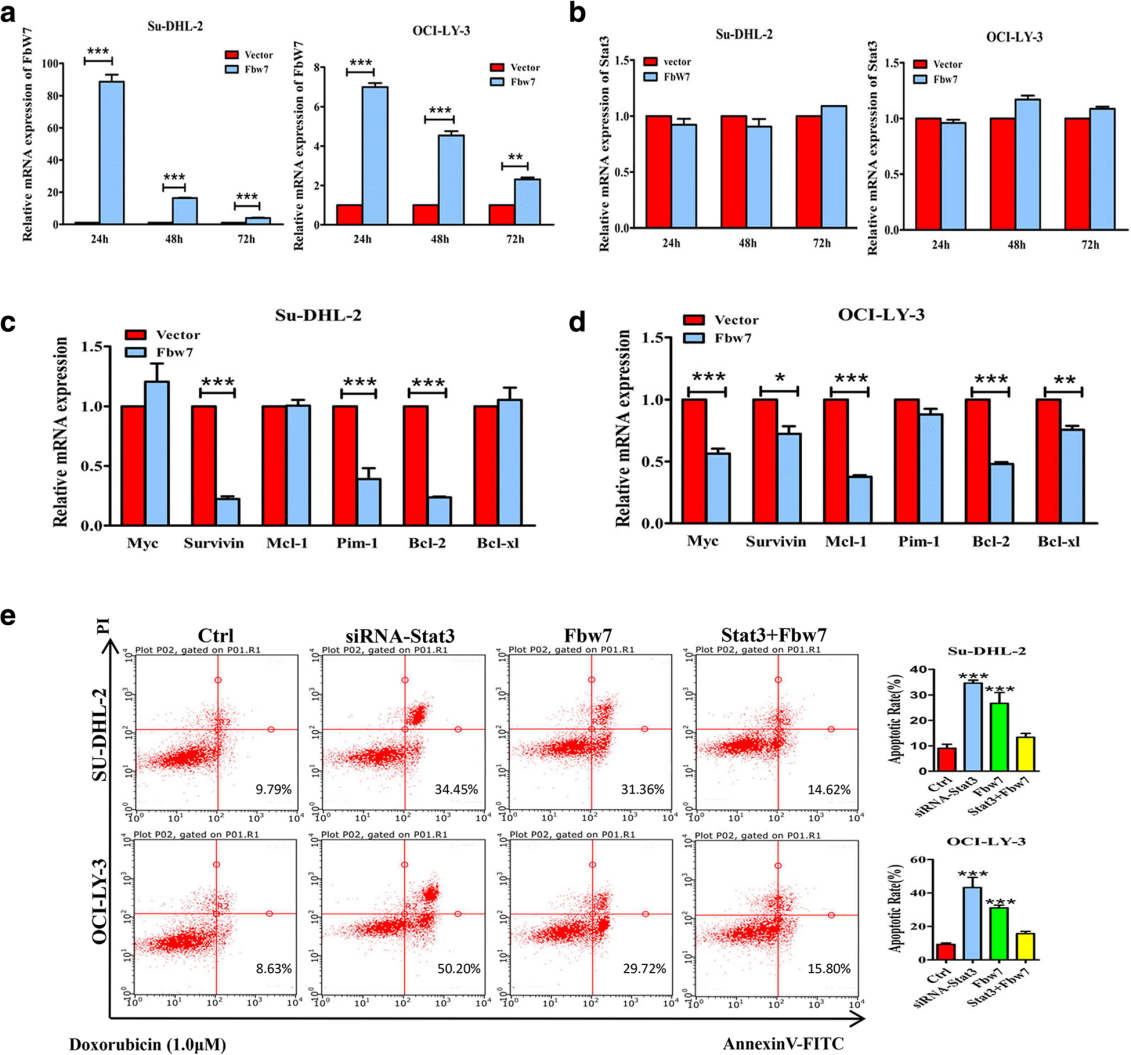
Fbw7 inhibits downstream Stat3 target genes that block apoptosis. **a-d,** qPCR analysis of the relative mRNA expression in ABC-DLBCL cell lines at the indicated time intervals. mRNA levels were normalized to the geometric mean of the housekeeping gene β-actin and calculated using the 2^−ΔΔCT^ method. The experiments were repeated three times. Statistical analysis was performed using a two-tailed unpaired Student *t* test.*, *P <* 0.05; **, *P <* 0.01; ***, *P <* 0.001. **a** (*right*), cells were transfected with Fbw7 and compared with vector control to detect Fbw7 expression. **b** (*left*), cells were transfected with Fbw7 and compared with vector control to detect Stat3 expression. **c** and **d,** qPCR analysis of the relative Myc, Survivin, Mcl-1, Pim-1, Bcl-2 and Bcl-xl mRNA levels after transfecting Fbw7 for 48 h in SU-DHL-2 and OCI-LY-3 cells, respectively. **e** siRNA of Stat3, plasmids of Fbw7 and Stat3 was transfected as instructed for 24 h in ABC-DLBCL cell lines. And then cell lines were cultured with Doxorubicin (1.0 μM) for 24 h to induce apoptosis before flow cytometry. Statistical significance was determined by a two-tailed, unpaired Student *t* test. ***, *P <* 0.001

Constitutively active Stat3 signaling leads to the upregulation of many downstream target genes in ABC-DLBCL, and our data indicate that Fbw7 could inhibit the expression of these genes through degradation of activated Stat3. To test this hypothesis, SU-DHL-2 and OCI-LY-3 cells were transduced with Fbw7 plasmid, and by qPCR showed upregulation of Fbw7 mRNA at the indicated time points (Fig. 5a). No significant change in Stat3 mRNA levels were detected (Fig. 5b), demonstrating that Fbw7 did not affect Stat3 mRNA. However, qPCR revealed that Fbw7 overexpression resulted in a downregulation of several Stat3 target genes that regulate apoptosis, in a cell-dependent manner (Fig. 5c). Results showed a significant reduction in Survivin, Pim-1 and Bcl-2 mRNA levels in SU-DHL-2 cells (right), as well as a significant reduction in Myc, Survivin, Mcl-1, Bcl-2 and Bcl-xl mRNA levels in OCI-LY-3 cells (left). Thus, our results suggest that Fbw7 promotes apoptosis by downregulating these anti-apoptotic genes in ABC-DLBCL.

To demonstrate whether Fbw7 promotes apoptosis through regulating Stat3, siRNA of Stat3, plasmids of Fbw7 and Stat3 was transfected as instructed in ABC-DLBCL cell lines, using Doxorubicin to induce apoptosis and staining with Annexin-V-PE followed by flow cytometry analysis. As predicted, SU-DHL-2 and OCI-LY-3 cell lines were more sensitive to apoptotic stimuli after transfecting siRNA of Stat3 or Fbw7 plasmid compared with control groups, and co-expression of Stat3 and Fbw7 plasmids reversed apoptosis compared with Fbw7 groups.

### Fbw7 expression is negatively correlated with pStat3^Tyr705^ levels in DLBCL patient samples

**Fig. 6:**
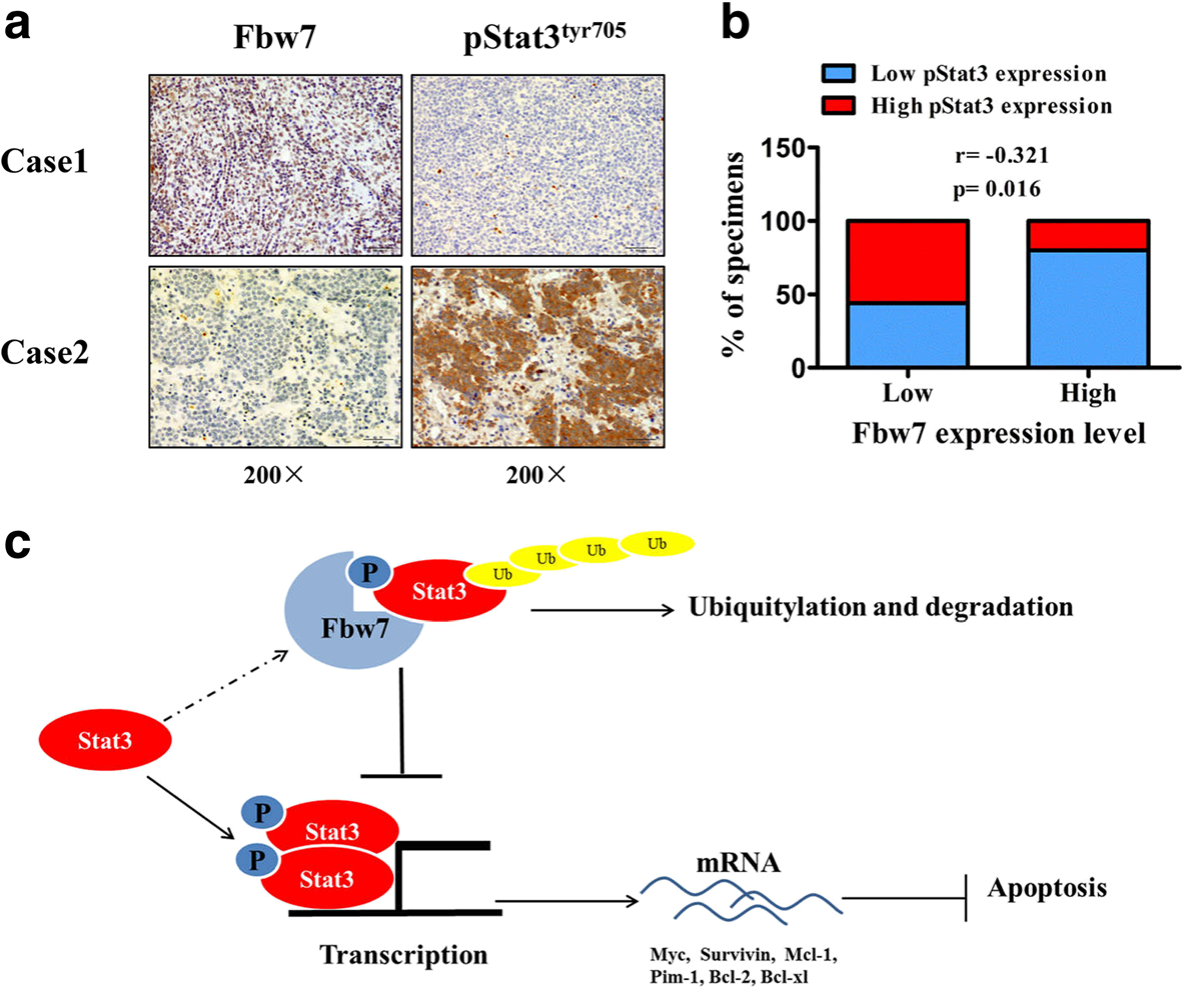
Fbw7 expression negatively correlates with Stat3 expression in DLBCL cell lines and clinical DLBCL samples. **a** IHC staining of Fbw7 and pStat3^Tyr705^ in human DLBCL tissues. Representative images of IHC staining from the same tumor samples are shown. **b** Spearman correlation analysis between Fbw7 and pStat3^Tyr705^ in 56 cases of DLBCL tissues. **c** schematic representation of the function and potential mechanism of Fbw7 in ABC-DLBCL

To further evaluate the relationship between Fbw7 and Stat3, the expression of Fbw7 and Stat3 was analyzed in 56 cases of DLBCL. IHC staining showed that high Fbw7 expression was associated with low pStat3^Tyr705^ expression in Case 1. Inversely, low Fbw7 expression was associated with high pStat3^Tyr705^ levels in Case 2 (Fig. 6a). Spearman rank correlation analysis further confirmed that Fbw7 expression was negatively associated with pStat3^Tyr705^ expression (Fig. 6b). These results demonstrated that high Fbw7 expression was associated with reduced levels of active Stat3 in human tumor samples, supporting our in vitro data.

## Discussion

The ubiquitin-ligase Fbw7 targets several proto-oncogenes for ubiquitination and degradation and acts as a tumor suppressor in many human malignancies. However, the Fbw7 substrates that have important roles in the development of specific cancers are unknown. Here, we investigated Fbw7 in ABC-DLBCL and found that Fbw7 targets Stat3 for ubiquitylation and degradation, and that Fbw7 inhibits downstream anti-apoptotic targets of Stat3.

DLBCL is attributable for nearly 40% of all non-Hodgkin lymphoma diagnoses and has been divided into the molecular subtypes GCB and ABC by genetic profiling. Overall survival is significantly reduced in the ABC-DLBCL compared with the GCB-DLBCL. In our study, we found that Fbw7 expression was reduced in DLBCL associated with the ABC subtype (Fig. 1b). Low Fbw7 expression also correlated with a poor prognosis (Fig. 1d). We also demonstrate that Fbw7 is an apoptosis regulator through flow cytometry analysis and cell viability assays (Fig. 2b and c). Previous studies have shown that Fbw7 targets c-Jun and Mcl-1, regulating apoptosis [18, 20], and these data interested us to investigate its molecular mechanism for regulating apoptosis in ABC-DLBCL.

Stat3, an important transcript factor in many human cancers, shows a high level of expression and activation in ABC-DLBCL [15]. Constitutive Stat3 activation is also a biomarker for poor prognosis after R-CHOP therapy [16]. Previous studies demonstrated Stat3 siRNA or kinase inhibitors reduced tumor proliferation in vitro [31]. These reports suggest targeting Stat3 could be a promising approach to therapy in ABC-DLBCL. Our data confirmed Fbw7 targets Stat3 for ubiquitylation and degradation, especially Stat3 phosphorylated at tyrosine 705. First, we found Fbw7 influenced the stability of Stat3 and pStat3^Tyr705^ in a dose-dependent manner (Fig. 3b). Further, we showed that Fbw7 interacts with Stat3 and pStat3^Tyr705^ in ABC-DLBCL cells by co-immunoprecipitation (Fig. 4a-c). Moreover, Fbw7 interacts with Stat3 and pStat3^Tyr705^ to regulate their ubiquitylation and degradation. It has been reported that Fbw7 binds to its substrates after they have been phosphorylated within conserved phospho-degron motifs, called Cdc4 phospho-degrons (CPDs) [32]. Several studies have demonstrated that Fbw7 targets phosphorylated substrates, including Myc, c-Jun, Mcl-1, Notch and KLF2 [17, 18, 20–22]. The ability of Fbw7 to degrade these oncogenes, specifically, makes it a tumor suppressor. Our results showed that Fbw7 interacts with and degrades Stat3 in ABC-DLBCL, and therefore; may be a viable target for Stat3-directed therapy.

Stat3 is required for tumor cell proliferation, infiltration, differentiation and apoptosis inhibition. Stat3 is activated by phosphorylation, which produces a molecule that spontaneously dimerizes, binds to DNA and activates transcription of downstream target genes [10, 11], some of which block apoptosis, including Myc, Survivin, Bcl-2, Mcl-1, Pim-1 and Bcl-xl [33–36]. Our results also revealed that Fbw7 overexpression reduced mRNA levels of these target genes in a cell-dependent manner in ABC-DLBCL cell lines. There was a significant reduction in Survivin, Pim-1 and Bcl-2 mRNA levels in SU-DHL-2 cells (Fig. 5c, right), as well as a significant reduction in Myc, Survivin, Mcl-1, Bcl-2 and Bcl-xl mRNA levels in OCI-LY-3 cells (Fig. 5c, left). Thus, both our clinical and experimental data suggest Fbw7 regulates Stat3 stability and downstream signaling, making it a promising target for therapy. Although it is well known that cyclin D1 is the target gene of Stat3 which regulates cell proliferation and cell cycle, qPCR revealed that Fbw7 overexpression did not result in significant reduction of cyclin D1 (Additional file 6). And the results of CCK8 proliferation assays and cell cycle did not show significant difference after Fbw7 overexpression in ABC-DLBCL cell lines (Additional file 4). It’s reported that only 2.1% of patients in total of 1435 express cyclin D1 in DLBCL [37]. Therefore our negative results of cyclin D1 may be related to its missing expression. Moreover, Fbw7-induced degradation of STAT3 is more important than other reported tumorigenesis including Myc, Notch, Jun, DEK and MCL1 in ABC-DLBCL (Additional file 7).

Fbw7 regulates a proliferative network that includes several oncogenes, and therefore, is considered a tumor suppressor in human cancers. There may be a viable approach for therapies directed at Fbw7 and its substrates. Studies have shown that Fbw7 expression is regulated by upstream proteins including p53, EBP2, Numb4, Pin1 and Hes-5 [38–41]. In addition, multiple microRNAs, including miR-25, miR-129-5p and miR-223 have also been demonstrated to regulate Fbw7 expression [42–44]. Together, these factors comprise a complex regulatory network that controls Fbw7 expression. However, exploiting this regulatory network for a means to restore or increase Fbw7 expression requires a deeper understanding of its transcriptional and post-transcriptional regulation. Further research into the upstream pathways that phosphorylate Stat3, marking it for Fbw7-mediated ubiquitylation, could also be a promising approach for drug discovery in ABC-DLBCL.

## Conclusions

In this study, we investigated the role of Fbw7 in ABC-DLBCL, and found it showed reduced expression in non-GCB DLBCL, which was associated with a poor outcome. Fbw7 expression promotes apoptosis in ABC-DLBCL cell lines. Furthermore, we demonstrated that Fbw7 targets Stat3 and especially activated Stat3 for ubiquitylation to regulate its stability. QPCR analysis also demonstrated that the downstream target genes of Stat3 which mediate anti-apoptotic effects were downregulated following Fbw7 expression in ABC-DLBCL. Together, our data clearly show that the ubiquitin-ligase Fbw7 targets Stat3 for ubiquitylation and degradation to regulate apoptosis in ABC-DLBCL, and our study may offer a promising approach for therapy by offering a new method of Stat3 inhibition.

## Additional files

Additional file 1: Correlation between Fbw7 expression and clinicpathological variables in 165 DLBCL cases. (DOCX 16 kb)
Additional file 2: Data of clinicpathological variables in 165 DLBCL cases. (XLS 314 kb)
Additional file 3: Primers for quantitative PCR. (DOCX 16 kb)
Additional file 4: Overexpression of Fbw7 did not inhibit proliferation in ABC-DLBCL cells. A, cell proliferation viability analysed by CCK8 assays. B, Flow-cytometry analyses of the cell cycle of the indicated ABC-DLBCL cells after transfecting Fbw7 for 48 h. Statistical analysis was performed using a two-tailed unpaired Student *t* test. (TIF 676 kb)
Additional file 5: Fbw7 interacts with Stat3 and pStat3^tyr705^ in HEK293T cells. A and B, Interaction between endogenous Fbw7 and Stat3 in HEK293T cells. Cell lysates were immunoprecipitated with anti-Fbw7, anti-Stat3 or anti-pStat3^Tyr705^ antibody followed by immunoblotting with anti-Fbw7, anti-Stat3 or anti-pStat3^Tyr705^, respectively. IgG was used as a control. (TIF 432 kb)
Additional file 6: Relative mRNA expression of cyclin D1. QPCR revealed that Fbw7 overexpression did not result in significant reduction of cyclin D1. (TIF 40 kb)
Additional file 7: Fbw7-induced degradation of STAT3 is more important than other reported tumorigenesis in ABC-DLBCL. A, western blotting showed overexpression of Fbw7 inhibit Stat3 more significant than other reported substrates of Fbw7 including Myc, Notch, Jun, DEK and MCL1. And the results of relative intensity were shown. B, Fbw7 decreases the stability of Stat3 more significant than other reported substrates of Fbw7 including Myc, Notch, Jun, DEK and MCL1. (TIF 680 kb)

## Abbreviations

ABC: Activated B-cell
DLBCL: Diffuse large B-cell lymphoma
FBS: Fetal bovine serum
GCB: Germinal center B-cell
HSC: Hematopoietic stem cell
IHC: Immunohistochemistry
NF-κB: Nuclear factor-κb
R-CHOP: Rituximab plus cyclophosphamide, doxorubicin, vincristine, and prednisone
Stat3: Signal transducer and activator of transcription 3
