# Supplementary material for: Fbw7 regulates apoptosis in activated B-cell like diffuse large B-cell lymphoma by targeting Stat3 for ubiquitylation and degradation
Source: J Exp Clin Cancer Res. 2017 Jan 10;36:10. doi: 10.1186/s13046-016-0476-y (PMC5223361; doi:10.1186/s13046-016-0476-y)
Supplement: Additional file 1: — Correlation between Fbw7 expression and clinicpathological variables in 165 DLBCL cases. (DOCX 16 kb) [file 13046_2016_476_MOESM1_ESM.docx]

**Supplementary Table 1. Correlation between Fbw7 expression and clinicopathological variables in 165 DLBCL cases**

| **Characteristics** | **All cases(N=165)** |  | **Fbw7 Expression (%)** | | |
| --- | --- | --- | --- | --- | --- |
|  |  | **Low (n=135)** | **High (n=30)** | **χ2 value** | ***p* value** |
| **DLBCL substype** |  |  |  | 14.154 | 0.000 |
| GCB | 43 | 27 | 16 |  |  |
| Non-GCB | 122 | 108 | 14 |  |  |
| **Age (years)** |  |  |  | 0.518 | 0.302 |
| ≤50 | 62 | 49 | 13 |  |  |
| >50 | 103 | 86 | 17 |  |  |
| **Sex** |  |  |  | 4.901 | 0.071 |
| Man | 91 | 69 | 22 |  |  |
| Woman | 74 | 66 | 8 |  |  |
| **EB virus** |  |  |  | 0.043 | 0.548 |
| Negative | 141 | 115 | 26 |  |  |
| Positive | 24 | 20 | 4 |  |  |
| **Tumor stage** |  |  |  | 6.877 | 0.076 |
| Ⅰ | 62 | 48 | 14 |  |  |
| Ⅱ | 32 | 30 | 2 |  |  |
| Ⅲ | 29 | 26 | 3 |  |  |
| Ⅳ | 42 | 31 | 11 |  |  |
