# Supplementary material for: Fbw7 regulates apoptosis in activated B-cell like diffuse large B-cell lymphoma by targeting Stat3 for ubiquitylation and degradation
Source: J Exp Clin Cancer Res. 2017 Jan 10;36:10. doi: 10.1186/s13046-016-0476-y (PMC5223361; doi:10.1186/s13046-016-0476-y)
Supplement: Additional file 3: — Primers for quantitative PCR. (DOCX 16 kb) [file 13046_2016_476_MOESM3_ESM.docx]

**Supplemental Table 2. Primers for quantitative PCR**

| Primer | Forward(5’-3’) | Reverse(5’-3’) |
| --- | --- | --- |
| Fbw7 | ACTGGGCTTGTACCATGTTCA | TGAGGTCCCCAAAAGTTGTTG |
| Stat3 | ACCAGCAGTATAGCCGCTTC | GCCACAATCCGGGCAATCT |
| β-actin | CATGTACGTTGCTATCCAGGC | CTCCTTAATGTCACGCACGAT |
| Myc | GTCAAGAGGCGAACACACAAC | TTGGACGGACAGGATGTATGC |
| Mcl-1 | TGCTTCGGAAACTGGACATCA | TAGCCACAAAGGCACCAAAAG |
| Pim-1 | GGCTCGGTCTACTCAGGCA | GGAAATCCGGTCCTTCTCCAC |
| Bcl-2 | GGTGGGGTCATGTGTGTGG | CGGTTCAGGTACTCAGTCATCC |
| Survivin | AGGACCACCGCATCTCTACAT | AAGTCTGGCTCGTTCTCAGTG |
| Bcl-xl | GAGCTGGTGGTTGACTTTCTC | TCCATCTCCGATTCAGTCCCT |
| Cyclin D1 | GCTGCGAAGTGGAAACCATC | CCTCCTTCTGCACACATTTGAA |
