# Supplementary figures and images for: Fbw7 regulates apoptosis in activated B-cell like diffuse large B-cell lymphoma by targeting Stat3 for ubiquitylation and degradation
Source: J Exp Clin Cancer Res. 2017 Jan 10;36:10. doi: 10.1186/s13046-016-0476-y (PMC5223361; doi:10.1186/s13046-016-0476-y)

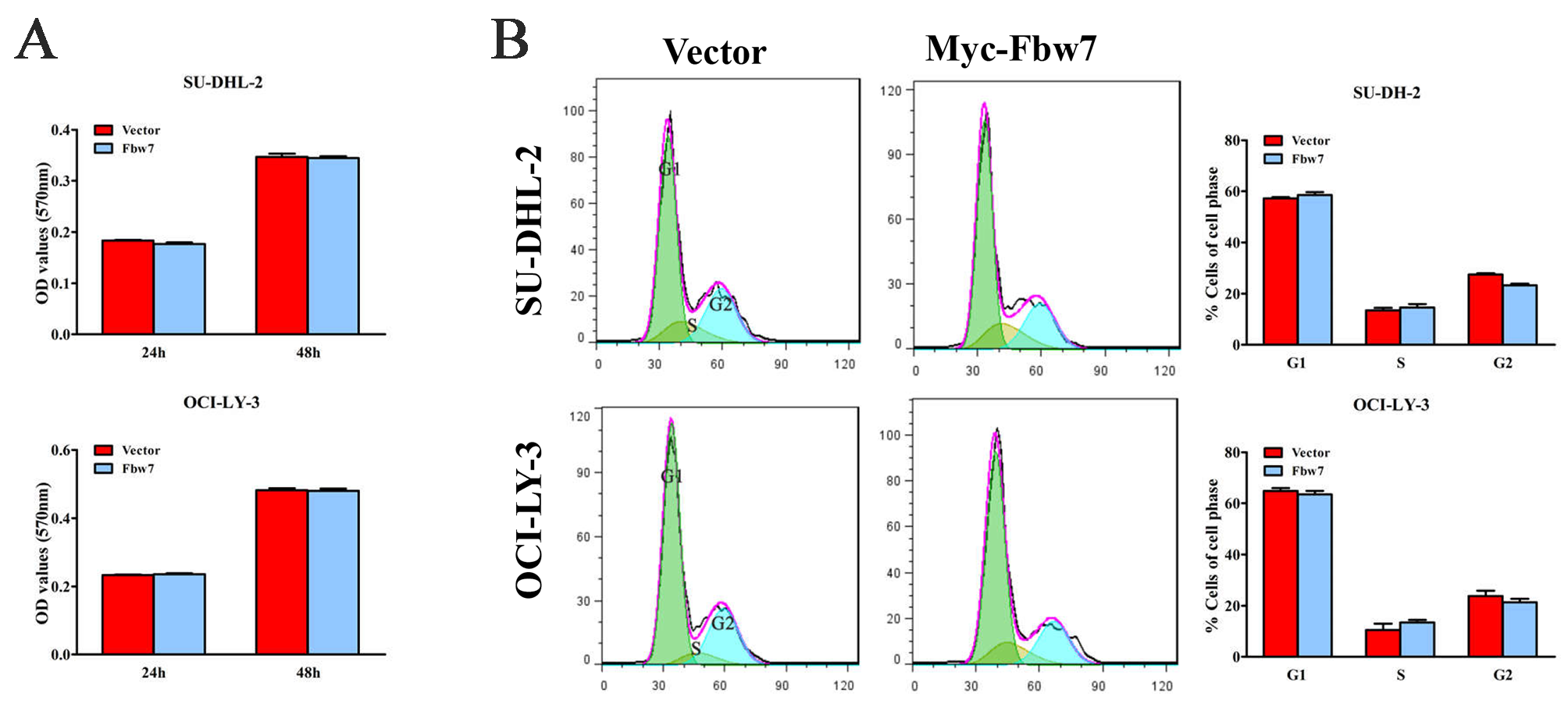

Supplement: Additional file 4: — Overexpression of Fbw7 did not inhibit proliferation in ABC-DLBCL cells. A, cell proliferation viability analysed by CCK8 assays. B, Flow-cytometry analyses of the cell cycle of the indicated ABC-DLBCL cells after transfecting Fbw7 for 48 h. Statistical analysis was performed using a two-tailed unpaired Student t test. (TIF 676 kb) [file 13046_2016_476_MOESM4_ESM.tif]

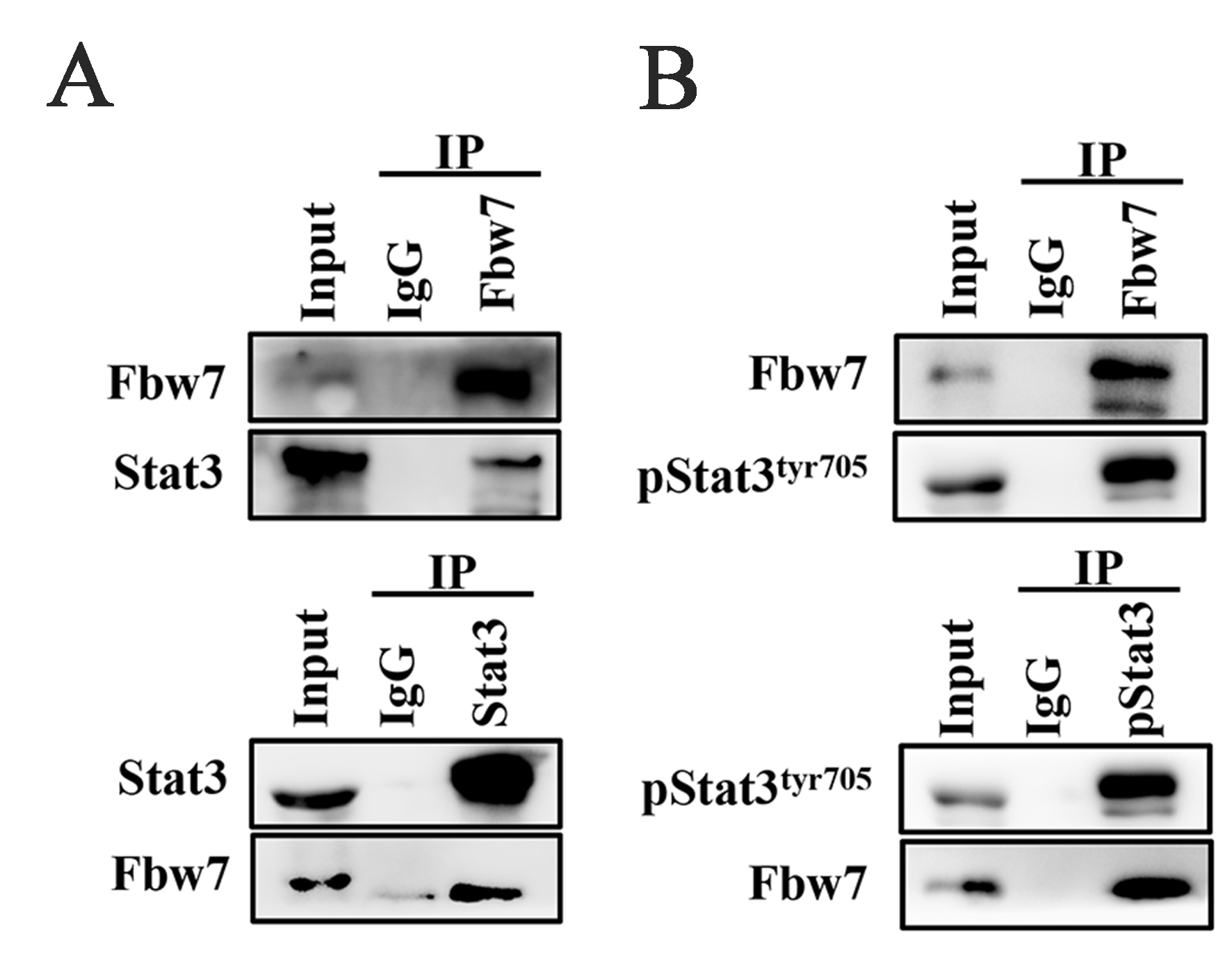

Supplement: Additional file 5: — Fbw7 interacts with Stat3 and pStat3tyr705 in HEK293T cells. A and B, Interaction between endogenous Fbw7 and Stat3 in HEK293T cells. Cell lysates were immunoprecipitated with anti-Fbw7, anti-Stat3 or anti-pStat3Tyr705 antibody followed by immunoblotting with anti-Fbw7, anti-Stat3 or anti-pStat3Tyr705, respectively. IgG was used as a control. (TIF 432 kb) [file 13046_2016_476_MOESM5_ESM.tif]

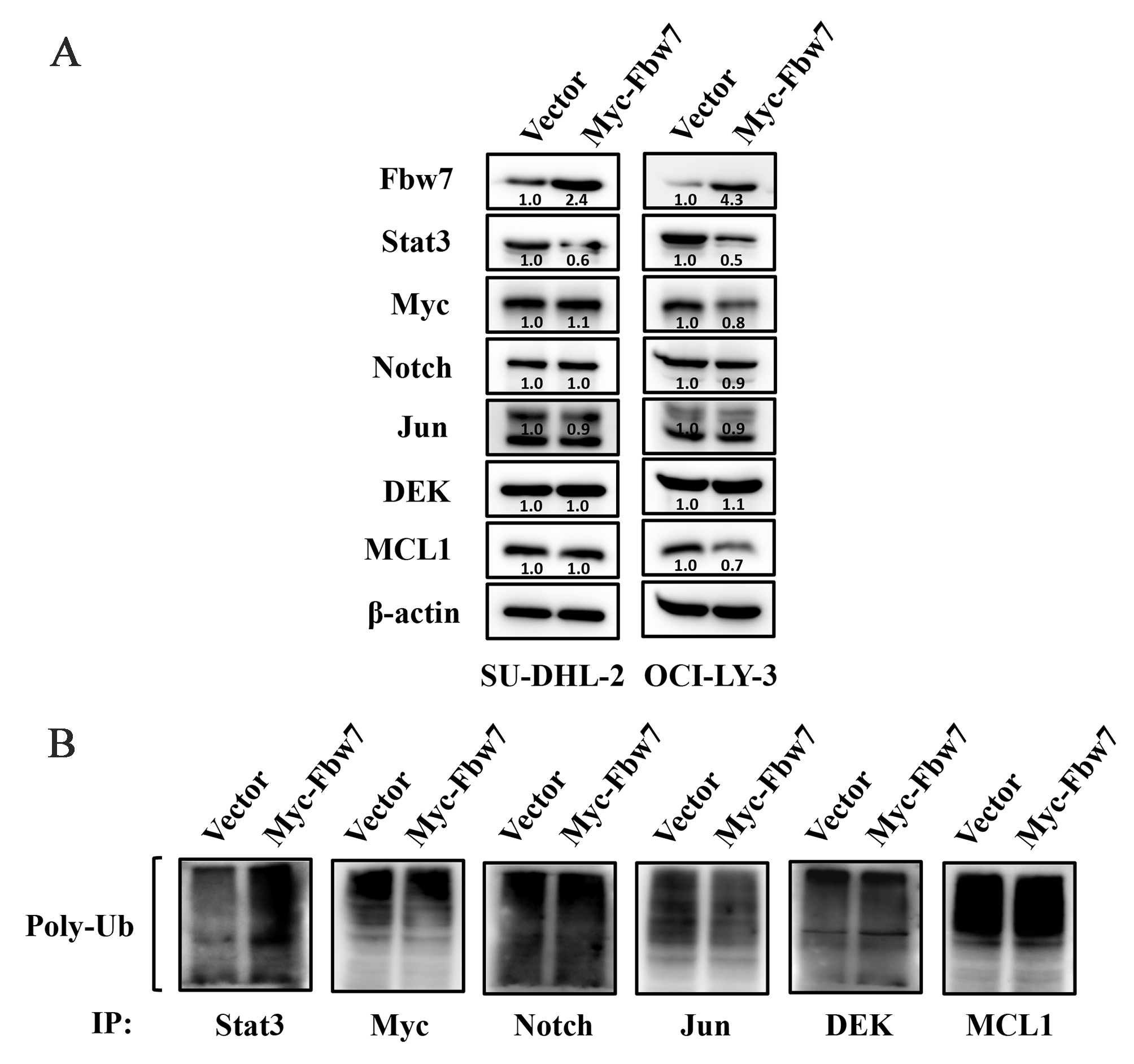

Supplement: Additional file 7: — Fbw7-induced degradation of STAT3 is more important than other reported tumorigenesis in ABC-DLBCL. A, western blotting showed overexpression of Fbw7 inhibit Stat3 more significant than other reported substrates of Fbw7 including Myc, Notch, Jun, DEK and MCL1. And the results of relative intensity were shown. B, Fbw7 decreases the stability of Stat3 more significant than other reported substrates of Fbw7 including Myc, Notch, Jun, DEK and MCL1. (TIF 680 kb) [file 13046_2016_476_MOESM7_ESM.tif]
